# An adaptive control framework based multi-modal information-driven dance composition model for musical robots

**DOI:** 10.3389/fnbot.2023.1270652

**Published:** 2023-10-09

**Authors:** Fumei Xu, Yu Xia, Xiaorun Wu

**Affiliations:** ^1^School of Music, Jiangxi Normal University, Nanchang, Jiangxi, China; ^2^School of Aviation Services and Music, Nanchang Hangkong University, Nanchang, Jiangxi, China; ^3^School of Information Engineering, Nanchang Hangkong University, Nanchang, Jiangxi, China

**Keywords:** CMAC, robot trajectory, multimodal information, robot dance, robot simulation

## Abstract

Currently, most robot dances are pre-compiled, the requirement of manual adjustment of relevant parameters and meta-action to change the dancing to another type of music would greatly reduce its function. To overcome the gap, this study proposed a dance composition model for mobile robots based on multimodal information. The model consists of three parts. (1) Extraction of multimodal information. The temporal structure feature method of structure analysis framework is used to divide audio music files into music structures; then, a hierarchical emotion detection framework is used to extract information (rhythm, emotion, tension, etc.) for each segmented music structure; calculating the safety of the current car and surrounding objects in motion; finally, extracting the stage color of the robot's location, corresponding to the relevant atmosphere emotions. (2) Initialize the dance library. Dance composition is divided into four categories based on the classification of music emotions; in addition, each type of dance composition is divided into skilled composition and general dance composition. (3) The total path length can be obtained by combining multimodal information based on different emotions, initial speeds, and music structure periods; then, target point planning can be carried out based on the specific dance composition selected. An adaptive control framework based on the Cerebellar Model Articulation Controller (CMAC) and compensation controllers is used to track the target point trajectory, and finally, the selected dance composition is formed. Mobile robot dance composition provides a new method and concept for humanoid robot dance composition.

## 1. Introduction

Robot dance is a highly attractive emerging research field. As an elegant and moving flexible art that perfectly combines auditory art and visual art, robot dance breaks through people's understanding of existing traditional entertainment methods and makes people's lives more colorful (Ros et al., 2014; Chen et al., 2019; Kobayashi et al., 2022). Music is usually related to motion, and this common assumption is based on many normal activities, such as spontaneously clapping our hands rhythmically while listening to a song, or swaying our heads and bodies in many ways along with the music (Santiago et al., 2012). Many studies support the analogy between music and movement. Robot dance composition is an important research field combining Robotics and human dance art (Tholley et al., 2012; Huang et al., 2021; Zhang and Li, 2022). Its research is not only conducive to the development of contemporary artificial intelligence but also helps to promote the rapid development of human-robot interaction. At the same time, robot dance has the ability to carry social services. Dancing which combines the rhythm, emotions, and tension of music is a beautiful, energetic, and natural art.

In recent decades, coordinated and creative robot behavior has been deeply studied (Liu et al., 2021; Song et al., 2021; Weigand et al., 2021). There are a large number of published research works on Humanoid robot dance. The framework of Oliviera et al. is based on the Lego Mindstorms NXT platform, trying to simulate dance behavior signals and generate corresponding behaviors by evaluating rhythm information from audio (Oliveira et al., 2008). Shinozaki et al. (2007) proposed an attempt to construct a robot dance system consisting of three units: dance unit sequence generation, dance unit database, and dance unit connection. They collaborated with a human dancer and recorded their dance performances. The selected actions and postures are copied onto the robot and stored in the database. Due to the need for connection between two dance movements, it is necessary to select a neutral position between the two dance units, so that the robot can perform any movement sequence. The work of Angulo et al. mainly focuses on the Sony Aibo animal robot platform (Angulo et al., 2011), with the goal of creating a system for interacting with users and generating a random movement sequence of the robot, thereby creating a human-machine interaction system. Driving the robot (steering, acceleration, etc.) through the obtained music information to express the elements conveyed by the music (Das et al., 2023; Wang et al., 2023). This method first obtains Musical Instrument Digital Interface (MIDI) data file data, segments the music file, clusters these blocks based on note blocks and turbulence values using a K-Means algorithm, and generates robot paths.

The mapping from sound to motion is not always predictable, and changes are often observed within a given task. There is no clear correspondence between movement and music interaction, and it is usually executed alternately with more interpretable gestures (Yu et al., 2021). Based on this assumption, Aucouturier et al. (2008) proposed a method for robots to perform free and independent dances to simulate synchronous and autonomous behavior between dynamic changes in human behavior (Zhou et al., 2017). For this purpose, a special type of chaotic dynamics is used, namely Chaotic Itrinerance (CI). CI is a relatively common feature in high-dimensional chaotic systems, which shows the cruise behavior between low-dimensional local Attractors through higher-dimensional chaos (Shim and Husbands, 2018; Zhang et al., 2019; Li et al., 2023). Compared to previous methods, it does not pre-arrange dance patterns or their alternations but rather builds on the dynamics of the robot and allows its behavior to appear in a seemingly autonomous manner (Yu et al., 2022). By converting the output of a neural network to generate real-time robot joint commands, the neural network processes pulse sequences corresponding to the dancing rhythm.

However, there are few researchers studying the composition of wheeled robot dance, and most of them only focus on the beauty of robot dance, neglecting an important factor in dance, which is dance composition (Taubes, 2000; Bryant et al., 2017; Jin et al., 2022; Su et al., 2023). Dance composition not only enhances the overall beauty of the dance but also is an essential factor in expressing the theme of the work. There are still some urgent problems to be solved in the composition of wheeled robot dance that is synchronized with music: (1) the key factors affecting dance composition are not clear; (2) there is no clear standard for the classification of dance composition types; and (3) there is no unified standard for evaluating the quality of robot dance composition.

**Figure 1:**
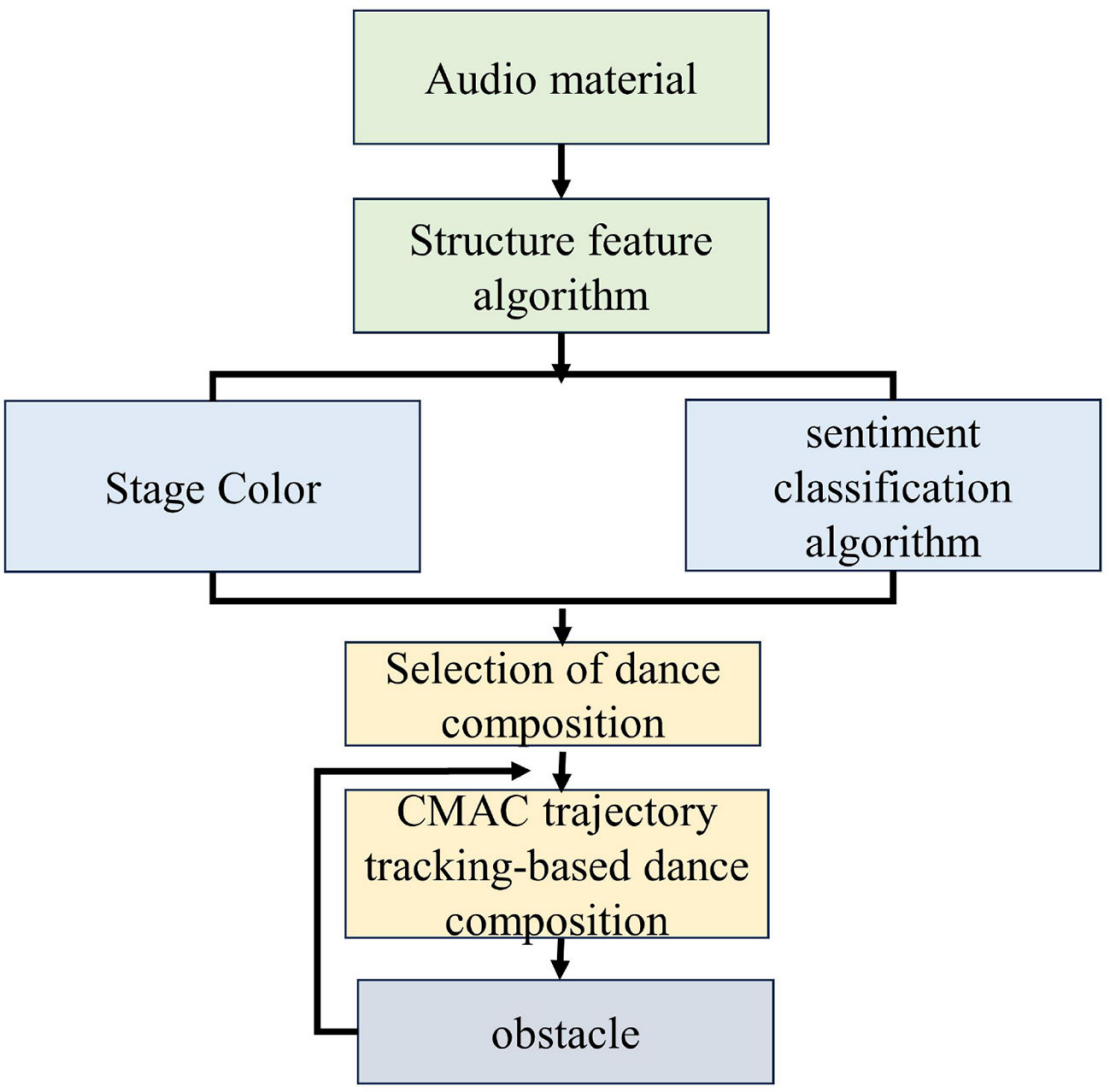
Process of robot dance composition system.

In order to overcome the shortcomings of the above research, this article focuses on the composition of mobile robot dance based on multimodal information. Music, as an indispensable element in dance, plays a significant role. In order to create a more beautiful composition in conjunction with music dance, music information is first analyzed, and then the corresponding composition of the dance is matched based on music information and other information. The research work includes the following aspects:

- (1) Music structure analysis: Using the Time Series Structure Feature music structure partitioning method, music is divided into several music segments based on its characteristics. The separated music segments are then integrated according to specific needs, and segments with a time of <5 sec are automatically added to the next music segment to obtain a corrected music structure.
- (2) Music sentiment analysis: Based on the analysis of music structure, a hierarchical sentiment detection framework is used to detect emotions in music using Thayer's two-dimensional sentiment model. Firstly, music emotions are divided into two groups based on different levels of tension. Then, based on the characteristics of timbre and rhythm, the two groups of emotions are further divided into two types of emotions, totaling four types.
- (3) Car information extraction: The E-pull wheeled robot used in the experimental simulation comes with a camera and distance sensor, which can easily obtain the ground color of the current stage where the car is located, as well as the real-time values of each distance sensor of the current car. Based on the size of the distance sensor values in each direction, the car movement can be guided.
- (4) Dance composition classification: Initialize the dance composition library and divide the dance composition into four categories corresponding to music emotions based on different emotions. Based on common sense, the dance compositions in each category are divided into two categories: one is the composition that the dancer dances well and loves the most, and the other is the composition that the dancer has the ability to dance. For the selected dance composition, an adaptive control framework based on the combination of CMAC (The Cerebellar model art controller) and compensation controller is used to track the trajectory of the dance composition and achieve the dance composition of the car. The workflow of the study is shown in Figure 1.

## 2. Method

### 2.1. A data model for mobile robots with non-holonomic constraints

**Figure 2:**
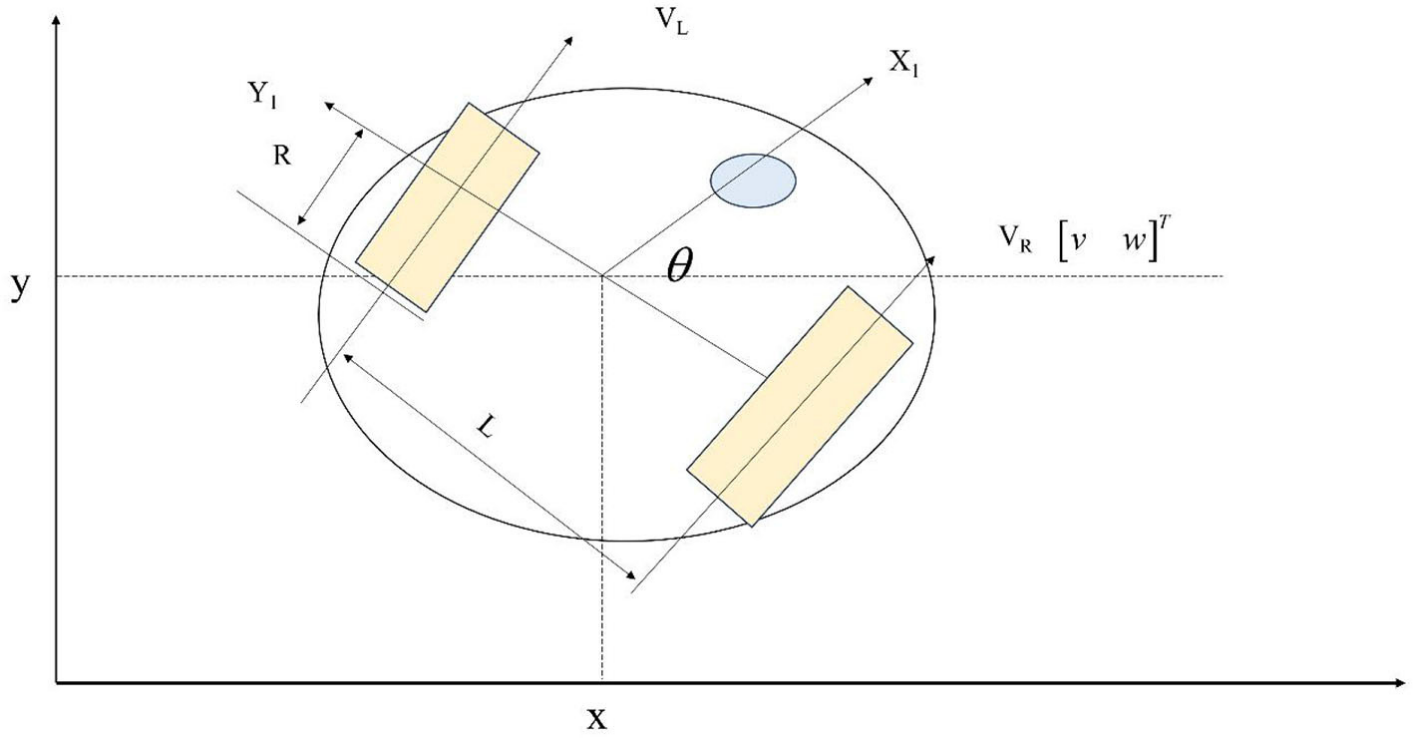
Mobile robots with non-holonomic constraints.

E-puck is a micro mobile robot, and its hardware and software are completely open source, providing low-level access for every electronic device and infinite scalability possibilities (Panwar et al., 2021). The E-puck is composed of a vehicle body and two driving wheels (Mansor et al., 2012). The structural diagram of the non-holonomic constrained mobile robot studied in this article is shown in Figure 2.

Representing the position coordinates of the non-holonomic constrained wheeled mobile robot as (x, y) in Figure 2, which is also the Cartesian coordinates of the centroid of the non-holonomic constrained mobile robot. The angle formed between the direction of the mobile robot's travel and the global coordinate system's X-axis is θθ, which is the direction angle of the mobile robot. V_L_ and V_R_ are the angular velocities of the left and right wheels, respectively. R is the radius of the wheel of the wheeled robot; L is the distance between the centers of the two driving wheels of the wheeled robot. Using $q={\begin{array}{ccc} {}[x & y & \theta ] \end{array}}^{T}$ to represent the current state of a non-holonomic constrained mobile robot, which is the pose of a wheeled robot. When the sliding of wheels of the wheeled mobile robot is not considered, its Kinematics equation can be expressed as:


(1) $$\begin{array}{l} \left[\begin{array}{c} ẋ\\ ẏ\\ \dot{\theta } \end{array}\right]=\left[\begin{array}{c} \frac{R}{2}\cos\theta \\ \frac{R}{2}\cos\theta \\ \frac{R}{L} \end{array}\begin{array}{c} \frac{R}{2}\cos\theta \\ \frac{R}{2}\cos\theta \\ \frac{R}{L} \end{array}\right]\left[\begin{array}{c} V_{L}\\ V_{R} \end{array}\right] \end{array}$$

Based on the relationship between the angular velocity of two wheels and the linear velocity and angular velocity w, it can be further concluded that:


(2) $$\begin{array}{l} \left[\begin{array}{c} ẋ\\ ẏ\\ \dot{\theta } \end{array}\right]=\left[\begin{array}{c} \cos\theta \\ \sin\theta \\ 0 \end{array}\begin{array}{c} 0\\ 0\\ 1 \end{array}\right]\left[\begin{array}{c} v\\ w \end{array}\right] \end{array}$$

In this article, it is specified that the angle unit of wheeled robots is radians, clockwise angle is positive direction, and counterclockwise angle is negative direction.

### 2.2. Multimodal information extraction

This study extracts information from Audio files. Firstly, the audio files are divided into musical structures, and then the musical beat, emotion, and tension of each structure are extracted.

**Figure 3:**
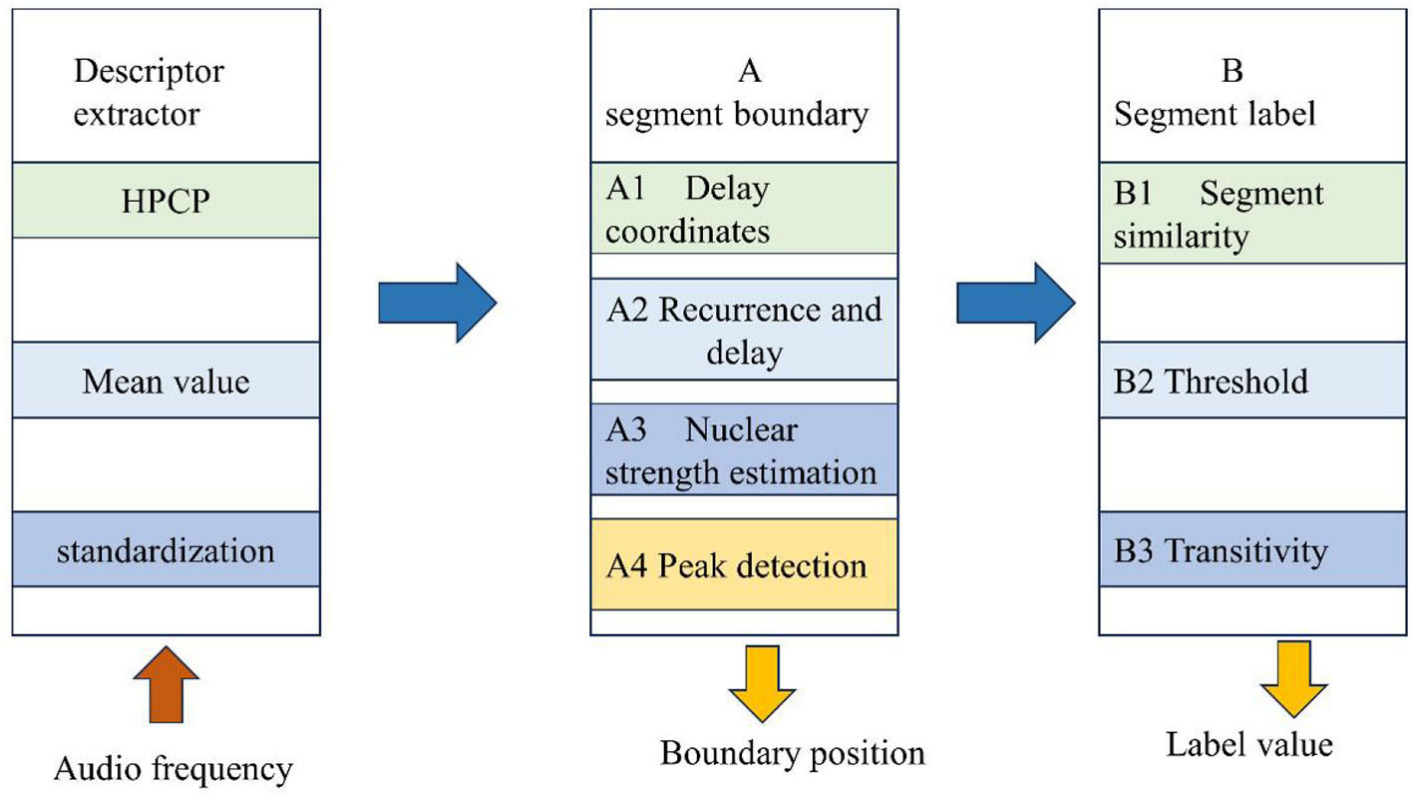
Framework of method.

Serrà et al. (2014) proposed a method based on a similar combination of structure features and time series. Structural features encapsulate local and global attributes of time series and allow the detection of boundaries between uniform, novel, or repetitive segments. Time series similarity is used to identify equivalent fragments corresponding to meaningful parts of music. Extensive testing with five benchmark music collections and seven different human annotations has shown that the proposed method is robust to different real data selections and parameter settings. Proposing a novel method for music structure annotation based on the combination of structural features and time series similarity (Xing and He, 2023). Before detecting segment boundaries and similarities, it is necessary to convert audio signals into feature representations that capture music-related information. For this purpose, pitch type (PCP) features, also known as chromaticity features, are used. PC functionality is related to many music retrieval tasks, especially widely used for music structure annotation. They typically use moving windows to calculate and generate multidimensional time series that capture the harmonic content of audio signals (Silva and Matos, 2022). Using HPCPs, enhanced PCPs that consider the presence of harmonic frequencies. In addition, hierarchical PCP (HPCP) reduces the impact of noise spectral components and is independent of tuning (Zhang and Tian, 2022). Using the same implementation and parameters as in the literature, it has 12 tone classes, a window length of 209 ms, and a skip size of 139 ms. Although choosing an enhanced version of PCP functionality, it is speculated that the proposed method is quite independent of the specific implementation details of the functionality. In fact, in the preliminary analysis, the so-called CENS chromaticity features were even correlated with the Mel frequency cepstrum coefficients (framework of the method is shown in Figure 3).

Usually, the dimensional approach adopts two theoretical psychological factors: stress (happiness/anxiety) and energy (calmness/energy), and divides musical emotions into four clusters: satisfaction, depression, vitality, and anxiety/fanaticism (Figure 5). As shown in the Figure 5, the definition of the four clusters is clear and distinguishable, and the two-dimensional structure also provides important hints for computational modeling. Based on Thayer's emotion hierarchical database model, a hierarchical framework for emotion detection is used. Intensity corresponds to “energy,” while timbre and rhythm correspond to “pressure.” Huron (1992) pointed out that among the two factors in the Thayer mood model (Zheng et al., 2020), energy is easier to calculate and can be estimated using simple amplitude-based measurements. Therefore, it is applied in emotion detection systems.

Therefore, firstly using features representing energy to classify all four emotions into two groups. If the energy is small, it is classified as the first group (satisfaction and depression); otherwise, classify it as Group 2 (lively and anxious). Then, use other characteristics to determine what the mood type is. This framework is consistent with the theory of music psychology. Meanwhile, as the performance of different features varies in distinguishing different emotional groups, this framework is conducive to using the most appropriate features for different tasks. In addition, like other hierarchical methods, it can better utilize sparse training data and its non-hierarchical correspondence.

**Figure 4:**
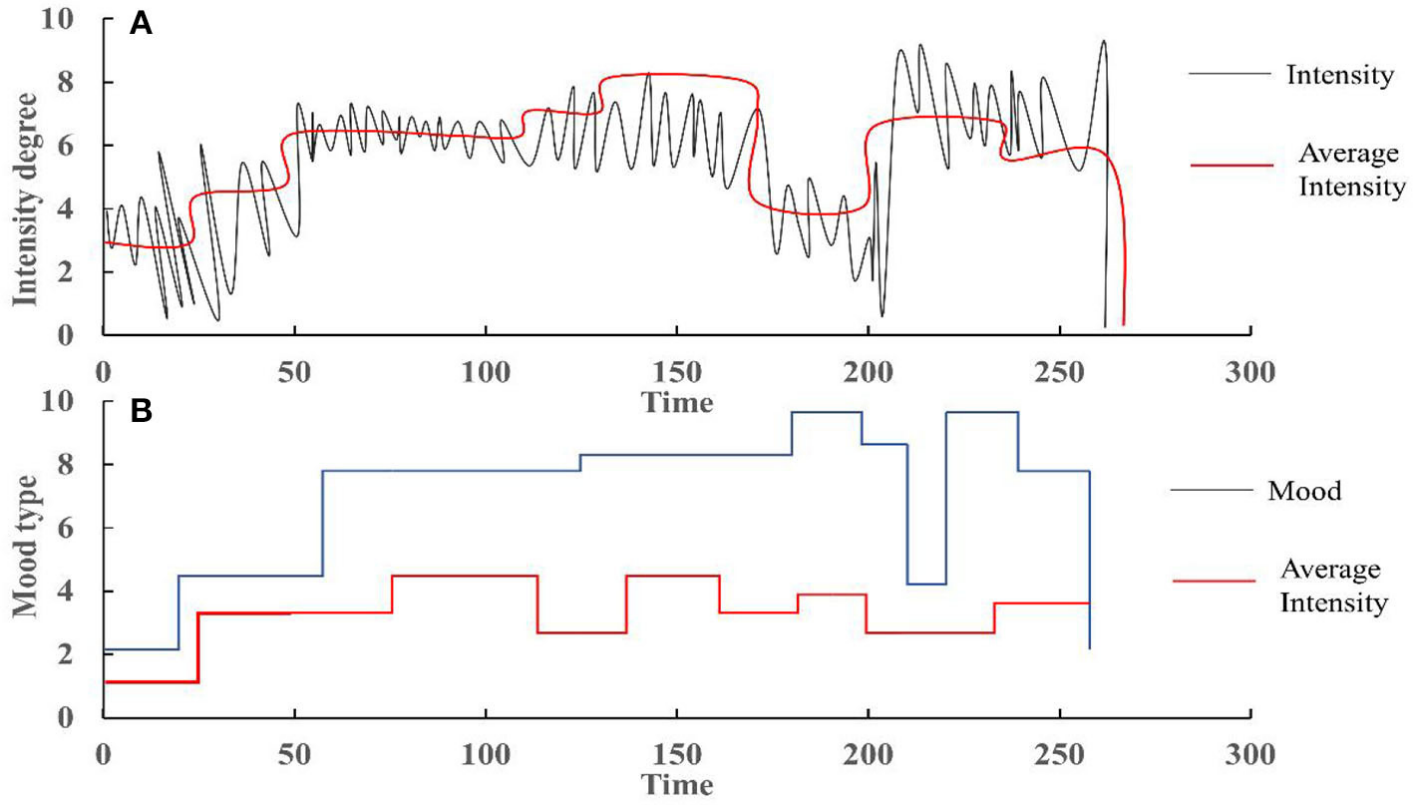
**(A)** Second tension and segmented tension; **(B)** emotions corresponding to music segments: 1: satisfaction, 2: depression, 3: vitality, 4: anxiety.

Based on these facts, it is usually best to set two weighting factors of 0.8 and 0.4 for the different importance of timbre and rhythm features in emotion detection in different emotion groups, as shown in Figure 4A, which shows the average tension per second and segment tension of music structure. Figure 4B shows the emotion categories of each music segment based on tension, timbre features, and rhythm features.

**Figure 5:**
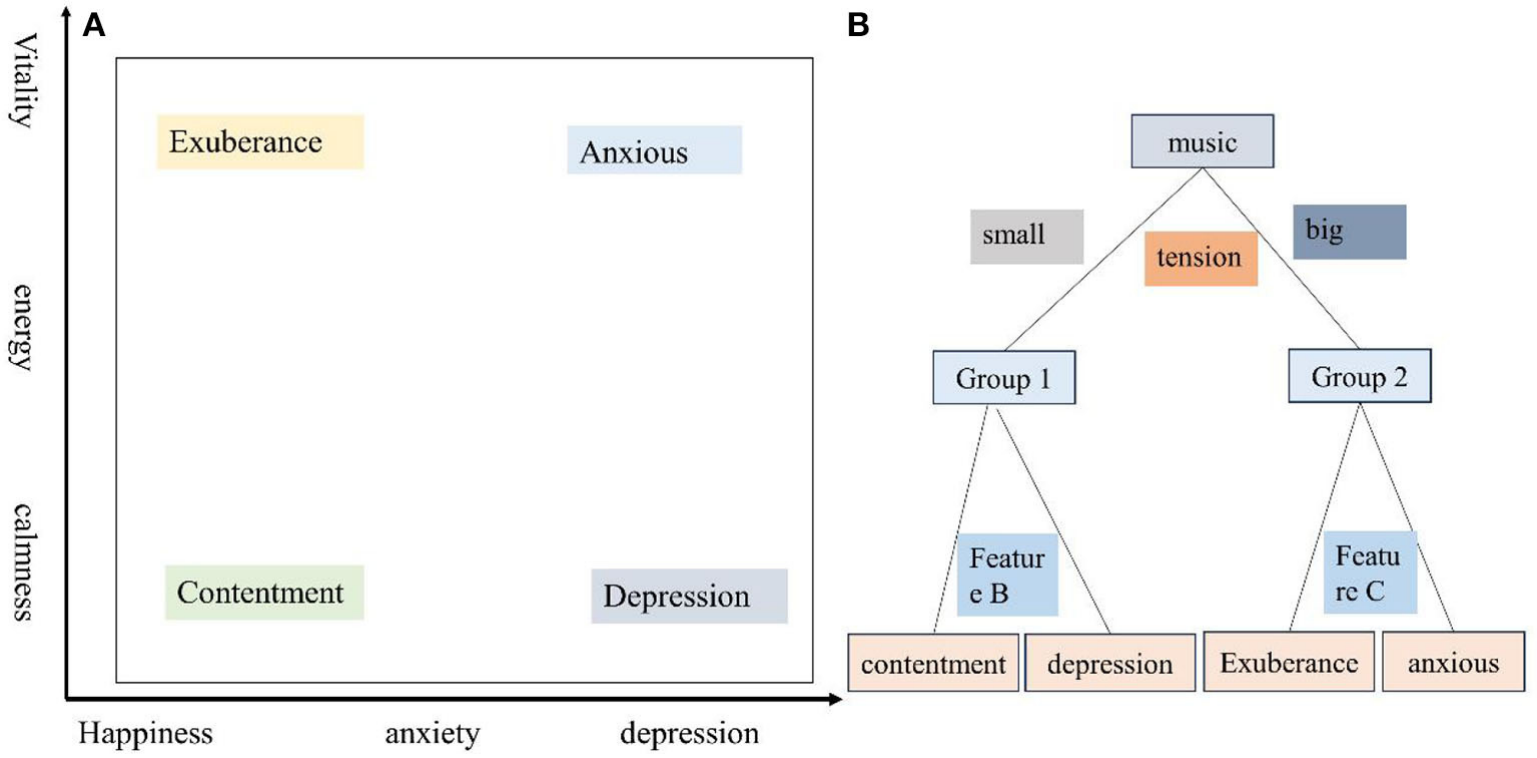
**(A)** Thayer's emotional model; **(B)** Layered emotion detection framework.

Set four colors on the stage floor to represent four emotions, as shown in Figure 5. The purpose of this design is to maintain consistency with our classification of music into four emotional categories: red represents satisfaction, blue represents anxiety, brown represents depression, and green represents vitality. When the robot car performs dance composition, extracting the current floor color serves as a guide for the robot composition. The E-puck robot car comes with a camera that can obtain an image of the area observed by the current camera. What we need is the stage color of the area where the current car is located. Therefore, we need to process the currently seen image.

Wb_ Camera_ Get_ The image() function reads the last image captured by the camera. Encode the image into a sequence of three bytes, representing the red, green, and blue levels of the pixel. Pixels are stored in horizontal lines from the top left side to the bottom right side of the image. Read the red, green, and blue pixels at the desired position, process the data, and compare it with the RGB values of the floor color to determine the color of the stage where the current car is located, and then determine its corresponding emotions. The formula for obtaining the current position pixel of the E-puck cart is as follows:


(3) $$\begin{array}{l} \begin{array}{c} image=wb\text{\_}camera\text{\_}get\text{\_}image\left(\right)\\ red\text{\_}pixel=image\left(position\text{\_}width,position\text{\_}height,1\right)\\ \begin{array}{c} green\text{\_}pixel=image\left(position\text{\_}width,position\text{\_}height,2\right)\\ blue\text{\_}pixel=image\left(position\text{\_}width,position\text{\_}height,3\right) \end{array} \end{array} \end{array}$$

Where position_weigth and position_heigtht take the position of the point. Due to the possibility of E-puck being located at the intersection of the A-color stage and the B-color stage, only a small portion of the A-color stage can be seen. Therefore, our target point should be selected in the lower right corner range of the image.

**Figure 6:**
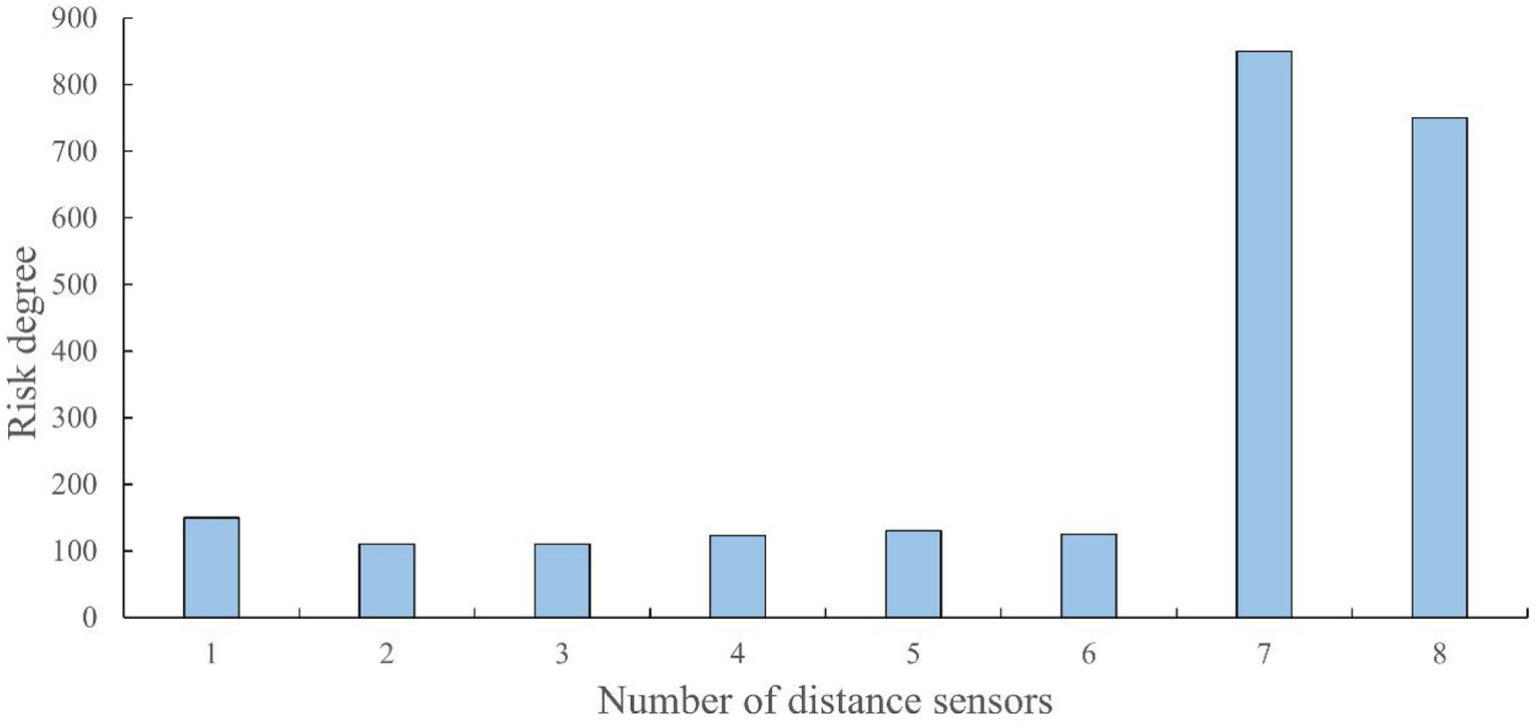
The relationship between risk and number of distance sensors.

When dancers dance on stage, they will automatically create a dance composition based on the size of the stage, thereby avoiding the embarrassment of falling off the stage. Based on this, a special square stage surrounded by four walls was constructed to ensure the safe performance of the E-puck dance composition on stage. When the current side encounters the wall, it turns toward a safe area without collision. To achieve this, collision detection and edge detection of the stage are required. The E-puck itself comes with 8 distance sensors, as shown in the following figure. When the car is moving, the 8 distance sensors can reflect whether it is safe in all directions at this time (Figure 6).

The x-axis represents the sensor numbers in 8 directions, while the y-axis direction represents the danger level of the sensor in this direction. The higher the value, the higher the danger level. Sensors 1–6 are located in a direction that is obstacle-free and safe, sensors 7–8 indicate that an obstacle in this direction is dangerous, sensors 0–200 indicate that the direction is safe, and sensors above 500 indicate that there is an obstacle in the direction. It is possible to consider changing the direction. When the distance is >1,000, it indicates that the obstacle is very close, and when it is close to 2,000, it indicates that it is dangerous and needs to be quickly changed.

## 3. Trajectory tracking based on CMAC

CMAC is composed of neurons that are locally adjusted and cover each other's receptive domains. It is a learning structure that simulates the human cerebellum. It is a local neural network model based on table query input and output and provides a multi-dimensional non-linear mapping capability from input to output. Lin and Peng (2004)proposed an adaptive control framework based on the combination of CMAC and compensation controller and used this method to track the trajectory of a machine car.

Consider third-order non-linear dynamic systems expressed in canonical form:


(4) $$\begin{array}{l} \{\begin{array}{c} x^{\left(n\right)}=f(x)+g(x)u(t)+d(t)\\ y=x \end{array} \end{array}$$

Where *f*(*x*) and *g*(*x*) are unknown real continuous functions (generally non-linear), *u*(*t*) and *y* is controlling input and out, respectively. d(t) is unknown external interference, $x={\left[x_{1},x_{2},...,x_{n}\right]}^{T}={\left[x,ẋ,...,x^{\left(n-1\right)}\right]}^{T}$ which is the state vector of the system assumed to be available.

The purpose is to design a control system so that state x can track a given reference trajectory $yd={\left[y_{d},y_{d},...,{y_{d}}^{\left(n-1\right)}\right]}^{T}$. The tracking error vector is defined as:


(5) $$\begin{array}{l} E≜{\left[e,ė,\ldots ,e^{\left(n-1\right)}\right]}^{T} \end{array}$$

*e*≜*y*_*d*_−*y* is a tracking error. If the dynamic characteristics and external disturbances are known [i.e., *f(x), g(x)*, and *d(t)* are known], the control problem can be solved through the so-called feedback linearization method. In this case, these functions are used to construct the ideal control law.


(6) $$\begin{array}{l} u^{*}=\frac{1}{g\left(x\right)}=\left[-f\left(x\right)-d\left(t\right)+{y_{d}}^{\left(n\right)}+K^{T}E\right] \end{array}$$

Where $K={\left[k_{n},...,k_{2},k_{1}\right]}^{T}$, *k*_*i*_ is a positive constant. Applying control rule Equation (6) to system Equation (4) resulted in the following dynamic errors:


(7) $$\begin{array}{l} e^{\left(n\right)}+k_{1}e^{\left(n-1\right)}+...+k_{n}e=0 \end{array}$$

Assuming the selection makes the polynomial $h\left(s\right)≜s^{n}+k_{1}s^{n-1}+...+k_{n}$$h\left(s\right)≜s^{n}+k_{1}s^{n-1}+\ldots +k_{n}$ all roots fall on the open left half of the complex plane. This means that for any initial condition asymptotically tracking the reference trajectory. However, functions *f(x)* and *g(x)* are usually not accurately known, and external interference is always unknown. Therefore, the ideal control law Equation (6) is not achievable in practical applications. In order to ensure that the system output *y* effectively follows the given reference trajectory *y*_*d*_, a CMAC-based adaptive monitoring system is proposed in the following sections.

Adaptive is based on the structure of a CMAC monitoring system, which combines a management controller and an adaptive CMAC. The control rule assumes the following form:


(8) $$\begin{array}{l} u=u_{A}+u_{s} \end{array}$$

Where *u*_*s*_ is compensation controller, *u*_*A*_ = *u*_*CMAC*_+*u*_*c*_ is adaptive CMAC. Compensation controllers can be designed separately to stabilize the state of the controlled system within a predetermined set of constraints. However, the control performance was overlooked. Therefore, adaptive CMAC is introduced to collaborate with compensation controllers to force system states within predefined constraint sets and achieve satisfactory tracking performance.

Assuming an optimal $\begin{array}{cc} CMAC & {u^{*}}_{CMAC} \end{array}$ exists to approximate ideal control law, let:


(9) $$\begin{array}{l} u^{*}={u^{*}}_{CMAC}\left(s,w^{*},m^{*},v^{*}\right)+\varepsilon =w^{*T}{\text{Γ}}^{*}+\varepsilon \end{array}$$

Where ε is minimum re-construction error, w^*^, m^*^, v^*^, Γ^*^ is optimal parameter of *w, m, v*, Γ, respectively. re-writing Equation (9):


(10) $$\begin{array}{l} u_{A}=u_{CMAC}\left(s,ŵ,\hat{m},\hat{v}\right)+u_{c}={ŵ}^{T}\hat{\text{Γ}}+u_{c} \end{array}$$

Where $ŵ,\hat{m},\hat{v},\hat{\Gamma }$ is optimal parameter estimation. Consider using Equation (4) to represent a non-linear dynamic system. The adaptive regulatory control rules based on CMAC are designed as Equation (8) Design Supervisory Control Rules, Equation (10) proposed adaptive CMAC. Here, in adaptive CMAC, the adaptive laws of CMAC are designed as Equations (11)–(15), and the compensation controller is designed as the estimation laws given in Equations (14), (15), where β_1_, β_2_, β_3_, and β_4_. It is a strictly positive constant. Therefore, the stability of the monitoring system based on CMAC can be guaranteed.


(11) $$\begin{array}{l} \{\begin{array}{c} \dot{\hat{w}}=\beta_{1}E^{T}PB_{m}\hat{\text{Γ}}\\ \dot{\hat{m}}=\beta_{2}E^{T}PBmC\hat{w}\\ \dot{\hat{v}}=\beta_{3}E^{T}PBmH\hat{w}\\ u_{C}=\hat{\delta }\;sgn(E^{T}PB_{m})\\ \dot{\hat{\delta }}=\beta_{4}\left|E^{T}PB_{m}\right| \end{array} \end{array}$$

## 4. Results and discussion

### 4.1. Simulation of mobile robot trajectory tracking system

**Figure 7:**
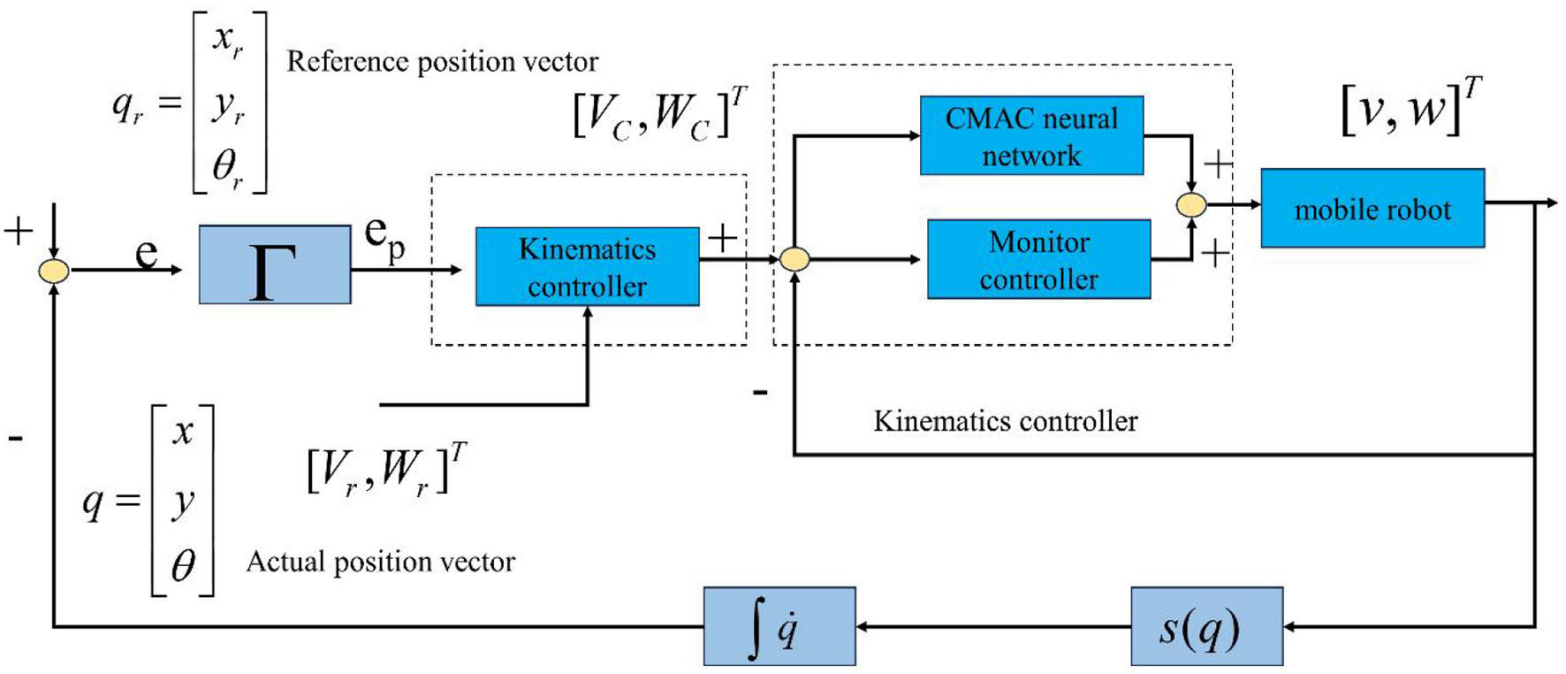
Framework of system control.

According to the Kinematics and dynamics model of mobile robots, the CMAC neural network is used to approximate and eliminate the non-linearity and uncertainty of the system, and an adaptive controller is used to compensate for the error of the CMAC neural network. The control structure of the designed mobile robot trajectory tracking system is shown in Figure 7.

Using $q_{r}={\left[x_{r},y_{r},\theta_{r}\right]}^{T}$ represents reference position vector, that is target trajectory, q = [*x, y*, θ]^*T*^ is the actual position vector, the tracking error in local coordinates can be expressed as:


(12) $$\begin{array}{l} e=\left[\begin{array}{c} e_{1}\\ e_{2}\\ e_{3} \end{array}\right]=T_{e}\left(q_{r}-q\right)=\left[\begin{array}{ccc} \cos\theta & \sin\theta & 0\\ -\sin\theta & \cos\theta & 0\\ 0 & 0 & 1 \end{array}\right]\times \left[\begin{array}{c} x_{r}-x\\ y_{r}-y\\ \theta_{r}-\theta \end{array}\right] \end{array}$$

The error change rate of mobile robots can be expressed as:


(13) $$\begin{array}{l} ė=\left[\begin{array}{c} {ė}_{1}\\ {ė}_{2}\\ {ė}_{3} \end{array}\right]=\left[\begin{array}{c} we_{2}-v+v_{r}\cos e_{3}\\ -we_{2}+v_{r}\sin e_{3}\\ w_{r}-w \end{array}\right] \end{array}$$

The speed control input used for tracking is represented as:


(14) $$\begin{array}{l} v_{c}=\left[\begin{array}{c} v_{r}\cos e_{3}+k_{1}e_{1}\\ w_{r}+k_{2}v_{r}e_{2}+k_{3}v_{r}\sin e_{3} \end{array}\right] \end{array}$$

Where *k*_1_, *k*_2_, *k*_3_ is designed parameter with >0. Controlling sign error is *e*_*c*_ = *v*_*c*_−*v*.

The incomplete mobile robot data model and its related parameters are shown in Table 1.

**Table 1:** E-puck robot parameters.

**Parameters**	**Values**
Mass	150 g
Moment of inertia	0.01 kg ·*m*^2^
Distance between wheels	70 mm
Radius of wheel	20.5 mm

**Figure 8:**
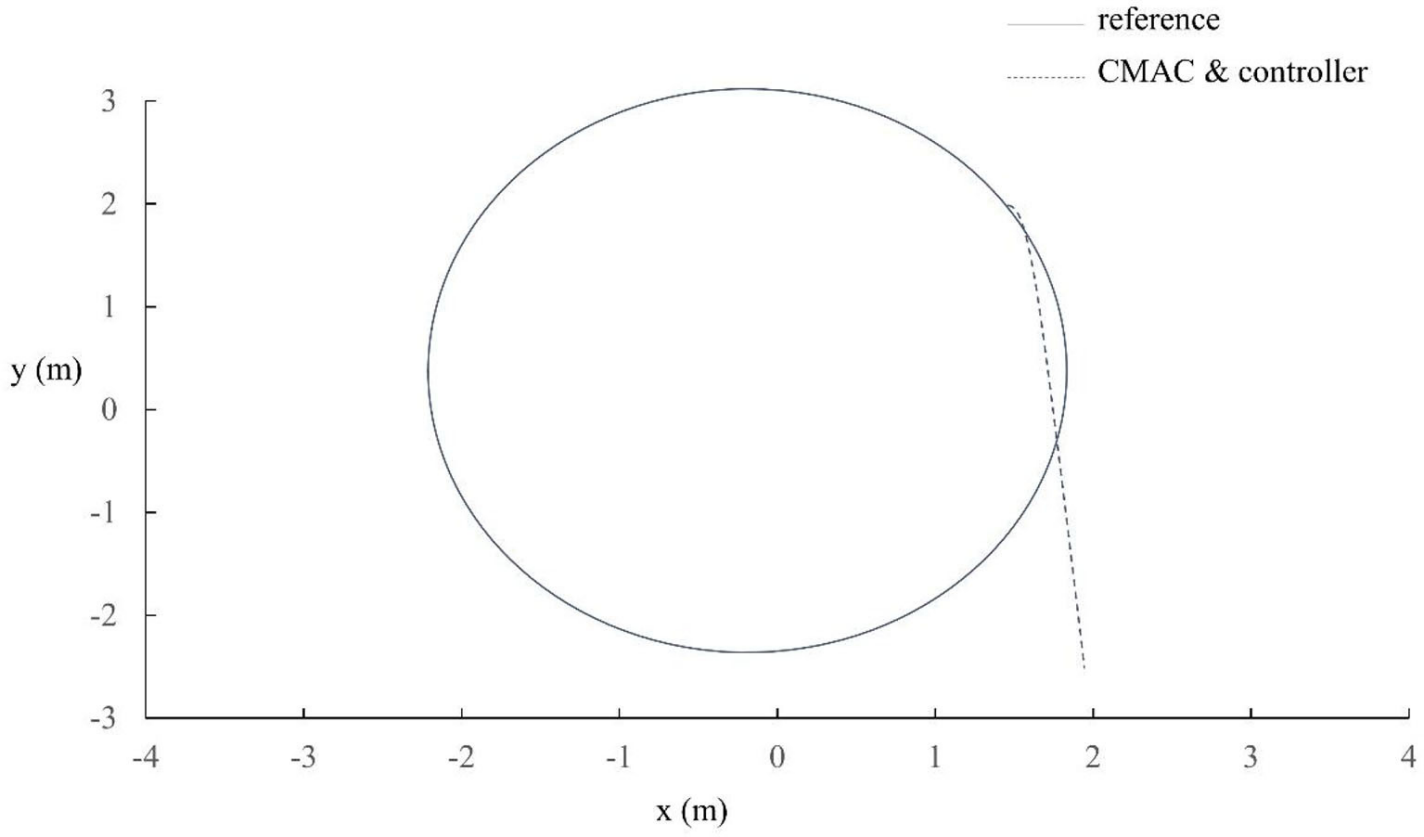
Track circle trajectory.

Selection of monitor controller parameters initialization, target initial pose $q_{r}={\left[-1,-0.7,\pi /2\right]}^{T}$, robot initial pose *q* = [−1.01, −1, π/2]^*T*^. *d* is external disturbance, assuming a square wave with an amplitude of ±0.5 and a 2π period. $g^{u}=g_{L}=1,K={\left[6,9,3\right]}^{T},Q=\left[\begin{array}{ccc} 24 & 21 & 5\\ 21 & 22 & 3\\ 5 & 3 & 2 \end{array}\right]$, $P=\left[\begin{array}{ccc} 9 & 7 & 2\\ 7 & 10 & 2\\ 2 & 2 & 1 \end{array}\right]$. Then parameters $\beta_{1}=5,\beta_{2}=\beta_{3}=0.75,\beta_{4}=0.01,\bar{V}=30,\delta =0.01,U_{w}=100,U_{m}=U_{v}=30$ are selected. In addition, CMAC parameters ρ = 4, *n*_*E*_ = 5, *n*_*B*_ = *n*_*R*_ = 2 × 4. Accepting field basis function set to $\phi_{ik}\left(s_{i}\right)=\exp\left[-{\left(s_{i}-m_{ik}\right)}^{2}/{\sigma^{2}}_{ik}\right]$ (i = 1, 2, 3 and k = 1,…,8), σ_*ik*_ = 4.8, [*m*_*i*1_, *m*_*i*2_, *m*_*i*3_, *m*_*i*4_, *m*_*i*5_, *m*_*i*6_, *m*_*i*7_, *m*_*i*8_] = [−8.4, −6, −3.6, −1.2, 1.2, 3.6, 6, 84]. Selecting the receiving field to cover the input space {[−6, 6], [−6, 6], [−6, 6]} and each input dimension. At the same time, the parameters use circular curves as reference trajectories for trajectory tracking and set the reference trajectory to *x*^2^+*y*^2^ = 4. The simulation results of trajectory tracking for mobile robots are shown in Figure 8.

### 4.2. Simulation of robot dance composition

Dance composition refers to the movement of points, lines, and planes that the dancer shows on stage. The lines and surfaces formed by these points are hidden, such as straight ahead, horizontal movement, walking in the shape of a circle, etc. The audience feels their existence—lines and shapes—from the imagery. As the Urelement of dance creation, the dots, lines, and planes in dance composition are full of vitality. As tools of art, they can enhance the power of action to convey emotions and convey ideas. Points, lines, and surfaces each have their own characteristics, and when using them, one should be good at choosing: according to the emotional needs expressed by music, the appropriate composition should be adopted. This section is divided into three parts: the first part is the integration of multimodal information; the division of the composition of the second part; the third part is the experiment and analysis after integrating the first two parts.

For the floor color (as shown in Table 2), obtaining the floor color where the machine trolley of the E-puck is located at the beginning of this music segment. When the root obtains the musical emotion of a music segment, the car obtains an initial speed based on the corresponding music emotion. Then, based on the stage color extracted by the car, the obtained initial speed is superimposed to obtain the true initial speed of the car (Table 3), and whether the current direction of travel is safe is obtained from the distance sensor.

**Table 2:** Initial speed of the car.

**Emotion**	**Contentment**	**Depression**	**Exuberance**	**Anxious**
Speed (m/s)	0.03	0.019	0.026	0.014

**Table 3:** The effect of stage color on initial speed.

**Emotion**	**Contentment**	**Depression**	**Exuberance**	**Anxious**
Speed (m/s)	0.014	−0.01	0.01	−0.005

Information integration:


(15) $$\begin{array}{l} in=\left[{x_{1}}^{T},...,{x_{n}}^{T}\right] \end{array}$$

Where *x*_*i*_ = [*e*_*i*_, *v*_*i*_, *v*_*ci*_, *s*], *i*∈[0, *n*], *n* is the number of music blocks, *e*_*i*_ is the emotion of the i-th musical block, *v*_*i*_ is car speed corresponding to the *i* musical block, and *v*_*ci*_ is the speed corresponding to the color of the stage where the car is located when starting the composition of the i-th music block, which affects the initial speed of the car, *s* represents distance sensor information.

**Figure 9:**
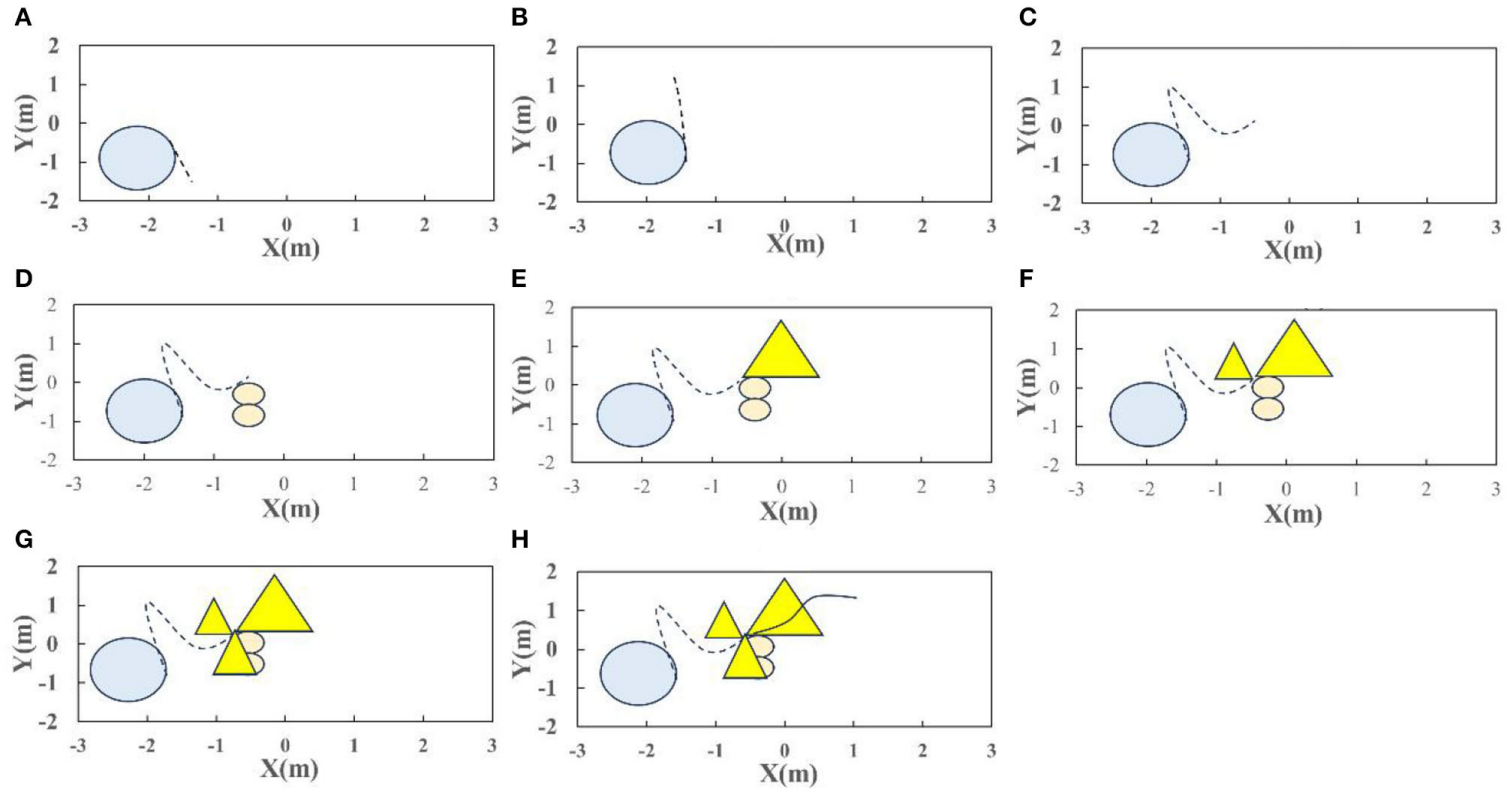
Robot dance composition. Composition of **(A)** circle, **(B)** line, **(C)** parabola, **(D)** Figure 8, **(E)** triangle, **(F)** triangle, **(G)** triangle, and **(H)** trigonometric curve.

According to emotional classification, dance composition is divided into four categories: satisfaction, frustration, vitality, and worry. Starting from our human emotions, for a drmer, each typeof dance has its best performing or favorite dance (Table 4). Based on this, divide each type of dance into two groups: favorite and ordinary. Based on stage information and distance sensor information, a partial dance composition is obtained, and some music segments are choreographed using multimodal information as shown in Figures 9A–H.

**Table 4:** Comparison with two classic robot dance compositions.

**Type**	**Or (2009)**	**Aucouturier et al. (2008)**	**Our method**
Diversity	1	0	1
Dynamicity	0	1	1
Stimulus source	Hearing	Hearing	Hearing, vision, tactile sensation
Real-time	0	0	1
Interactiveness	0	0	1
Extensibility	1	0	1
Musical information	Emotion	Rhythm	Multi-information
Predefine	1	0	0

Where “0” represents the method that has no capability, and “1” represents has corresponding capability.

For the above simulation results, when performing dance composition based on the current obtained music segment information and the information obtained by the car, the dance composition of a single music segment performed well. Although the dance composition of the next music segment and the music as a whole were not taken into account, it demonstrated the overall beauty of the dance composition. The disadvantage is that if several consecutive music clips have the same musical emotions, using simple matching and random methods to select dance compositions lacks interest and diversity. A more intelligent composition selection method is needed to solve this problem. Moreover, when the time of a music segment is very short, if the emotional matching of the segment music is a relatively complex composition (Figure 8 in the picture), the composition of the segment cannot express the beauty that we humans imagine. The simulation results show us the important position of dance composition in dance. A good dance composition can increase the appeal of music, better reflect the emotions expressed by a certain music segment, and inject soul into the entire dance, giving people a sense of beauty.

The unexpected purpose of the framework structure proposed in this article is to address the limitations of current robot dance composition, such as (1) music and composition are predefined, and the diversity and fun of dance composition cannot be guaranteed; (2) the music used in dance composition lacks intrinsic information; (3) There was no interaction with the outside during the robot dance process. The following is a comparison between the proposed framework and two classic robot dance composition studies.

### 4.3. Robustness of trajectory tracking

**Figure 10:**
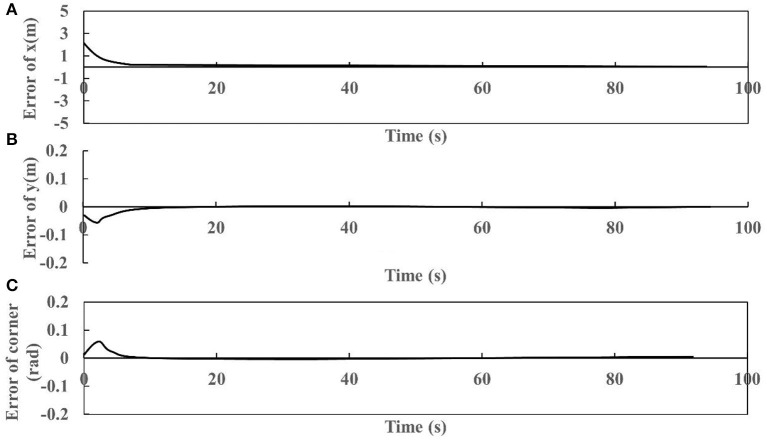
Robot trajectory tracking error of x **(A)**, y **(B)**, and corner **(C)**.

The robustness of robot trajectory tracking was tested by setting the parameters of the robot, and the results are shown in Figure 10. We can find from Figure 10 that the robot has a significant robustness in trajectory tracking. Specifically, the error rate of directions x and y, and the corner would be zero after 15s. The fact that the robot tracking error tends to zero proves that the system is stable.

### 4.4. Prospect

Dance is a moving, flexible art, and composition is to express the thoughts of the characters and the theme of music according to the lines of actors' actions on the stage. Dance composition is the soul of future robot dance, and dance without composition is incomplete. Studying robot dance composition is beneficial for the development of the robot field and is a need for a social and spiritual civilization.

This article explores the entire process of dance composition for wheeled robots based on multimodal information, dividing music into several music structures according to the Time Series Structure Feature method. Then, a hierarchical music emotion analysis method is used to detect the music emotions of each segment, and adopt stage color and distance sensor information. The core work is multimodal information acquisition and dance composition, and there are still many areas that need improvement in the next step of research:

- (1) In this paper, the composition of wheeled robot dance is divided into musical structures, and then dance composition is carried out according to different emotions of different structures, in which environmental information such as music and stage is taken into account, but there are many other details related to on-site emotions that are not taken into account, such as beat information in music clips, Stage lighting features, etc.
- (2) Only using an adaptive control framework based on CMAC and compensation controller for trajectory tracking in dance composition is not perfect for dance composition. The graphics that can be traced based on the framework proposed in this article are only limited to those that can be clearly expressed using formulas. In stage composition, there are still many complex compositions that cannot be expressed using simple formulas. Further research is needed to find suitable path-tracking algorithms for these types of graphics.
- (3) For the division of music emotions, a relatively rough emotion classification algorithm is used in the model, which is only used for simple connections within the model. However, it is necessary to improve it in future work, expand classification categories, and improve its classification accuracy.

## 5. Conclusion

This study proposed a dance composition model for mobile robots based on multimodal information. Music, as a key factor in robot dance composition, is first divided into several music structures according to the Time Series Structure Feature method; then, a hierarchical music emotion analysis method is used to detect the music emotions of each segment; obtain the stage color of the current car on the stage, and use the stage color as a form of interaction with the audience. Different colors represent the current mood or atmosphere of the audience, which can affect the starting speed of the car. Finally, the distance sensor provided by the car detects the safety of the distance between surrounding objects and the car, in order to remind the car of safe movement.

On the basis of obtaining multimodal information, and integrating multimodal information, there are four types of compositions: satisfaction, depression, vitality, and worry. Each composition is further divided into small car special hobby compositions and general hobby compositions. When matching with multimodal information, a composition class that matches music emotions is selected based on emotions, and then a random matching is performed on this composition class to select a composition. When selecting a composition, the total path length is first calculated based on the initial speed and the time length of the current music segment. The target point is determined based on the selected composition. An adaptive control framework based on CMAC and compensation controller is used for dance composition trajectory tracking. Finally, the wheeled robot constructs the selected dance composition. The experimental results indicate that the model can successfully achieve dance composition for mobile robots.

This study mainly contributed to (1) proposing a new mobile robot dance composition framework. This study is based on the traditional framework of music information-driven robot dance, proposing to first divide music into structures, and then analyze the music emotions for each music structure. Then, based on this information, a dance composition is selected. For the planned composition, an adaptive control framework based on CMAC and compensation controller is used to track the trajectory of the dance composition, achieving the dance composition of the car; (2) using an adaptive control framework based on CMAC and compensation controller for trajectory tracking to form a robot dance composition. This paper adopts an intelligent method to perform dance composition on mobile robots. Simply select the dance composition corresponding to the music's emotion, and different specifications of graphics can be constructed based on the size of the music segment time, which has higher flexibility, interest, and viewing value.

As an important branch of humanoid robot dance, mobile robot dance composition has laid a solid foundation for the future development of humanoid robot dance and provides a new method and idea for humanoid robot dance composition.

## Data availability statement

The original contributions presented in the study are included in the article/supplementary material, further inquiries can be directed to the corresponding author.

## Author contributions

FX: Conceptualization, Methodology, Validation, Writing—original draft. YX: Visualization, Writing—review and editing. XW: Funding acquisition, Project administration, Supervision, Writing—review and editing.
